# Ultrasonography Comparison of Diaphragm Morphological Structure and Function in Young and Middle-Aged Subjects with and without Non-Specific Chronic Low Back Pain: A Case-Control Study

**DOI:** 10.1155/2022/7929982

**Published:** 2022-12-16

**Authors:** Wenwu Xiao, Fuming Zheng, Ke Dong, Zhuangfu Wang, Yao Zu, Chuhuai Wang

**Affiliations:** ^1^Department of Rehabilitation Medicine, The First Affiliated Hospital, Sun Yat-Sen University, Guangzhou 510080, China; ^2^Department of Rehabilitation Medicine, The Third Affiliated Hospital, Sun Yat-Sen University, Guangzhou 510080, China

## Abstract

**Background:**

It is reported that impaired postural control in patients with non-specific chronic low back pain (NCLBP) was associated with “core” trunk muscle incoordination. However, as the diaphragm is an important component of the “core” deep trunk muscle group, we still know little about the potential relationship between diaphragm dysfunction and NCLBP.

**Objectives:**

This case-control study is intended to investigate the changes of diaphragm morphological structure and function in young and middle-aged subjects with and without NCLBP by ultrasound evaluation and its possible validity in predicating the occurrence of NCLBP.

**Methods:**

31 subjects with NCLBP (NCLBP group) and 32 matched healthy controls (HC group) were enrolled in this study. The diaphragm thickness at the end of inspiration (*T*^ins^) or expiration (*T*^exp^) during deep breathing was measured through B-mode ultrasound, and the diaphragm excursion (*T*^exc^) was estimated at deep breathing through M-mode ultrasound. The diaphragm thickness change rate (*T*^rate^) was calculated by the formula: *T*^rate^=(*T*^ins^ − *T*^exp^)/*T*^exp^ × 100%.

**Results:**

Compared with the HC group, the NCLBP group had a significant smaller degree of T^ins^ (*t* = −3.90, *P* < 0.001), *T*^exp^ (*Z* = −2.79, *P*=0.005), and *T*^rate^ (*t* = −2.03, *P*=0.047). However, there was no statistical difference in *T*^exc^ between the two groups (*t* = −1.42, *P*=0.161). The binary logistic regression analysis indicated that *T*^rate^ (OR = 16.038, *P*=0.014) and *T*^exp^ (OR = 7.714, *P*=0.004) were potential risk factors for the occurrence of NCLBP.

**Conclusions:**

The diaphragm morphological structure and function were changed in young and middle-aged subjects with NCLBP, while the diaphragm thickness change rate (*T*^rate^) and diaphragm thickness at the end of expiration (*T*^exp^) may be conductive to the occurrence of NCLBP. Furthermore, these findings may suggest that abnormal diaphragm reeducation is necessary for the rehabilitation of patients with NCLBP.

## 1. Introduction

Low back pain is a leading contributor to global disease burden [1, 2], which is the first leading cause of years lived with disability (YLDs) [3]. Approximately 90% of chronic low back pain are unclear etiologically and lack effective therapies [4, 5], which is deemed as non-specific chronic low back pain (NCLBP) [4, 6]. It is worth noting that the epidemiological evidence shows a rising incidence of NCLBP among young and middle-aged people [1].

An increasing number of studies suggested that the functional incoordination of the “core” trunk muscles and the postural control disorder were the important causes of low back pain [7, 8]. The diaphragm muscle, as one of the important components of the “core” deep trunk muscles as well as the main respiratory muscle, plays a key role in both respiratory and postural control [9–11]. However, whether there is a potential relationship between diaphragm dysfunction and low back pain is rarely reported, and the pathogenic mechanisms involved in it remain poorly understood.

From a clinical treatment perspective, several previous studies have validated that diaphragm exercise could effectively relieve the pain symptoms, strengthen muscle activity, enhance body stability, and increase reliance on back proprioceptive signals in patients with low back pain [12, 13]. In addition, previous studies also have manifested that diaphragm of low back pain patients is vulnerable to fatigue [14] and the characteristics of that is altered [15, 16]. In conclusion, these studies indicated that if the human body could not balance the diaphragm breathing demands and postural control, there would be destabilization of the spine, which leads to low back pain [17]. Besides, it also revealed that the diaphragm morphological structure and function might be changed in low back pain patients.

Ultrasound is a convenient and reliable tool to measure the static and dynamic diaphragm morphological structure and function during breathing [16, 18–20]. However, the application of diaphragm ultrasound in subjects with low back pain is seldom, and the results of relevant studies are inconsistent, i.e., no significant change of diaphragm thickness and excursion between NCLBP and asymptomatic subjects in one study [16], while the thinner diaphragm thickness in patient with lumbopelvic pain compared with asymptomatic subjects in another study [21]. Therefore, it deserves to future explore the changes of diaphragm morphological structure and function and the possible pathogenic mechanisms of that in subjects.

Above all, this case-control study is designed to investigate the diaphragm morphological and functional changes in patients with NCLBP and its potential role in the development of NCLBP by ultrasound evaluation. Through this endeavor, the study would shed light on revealing the etiology of NCLBP, optimizing rehabilitation treatment and drawing more clinical attention of NCLBP.

## 2. Methods

### 2.1. Study Design and Settings

This case-control study was approved and supervised by the Ethics Committee of the First Affiliated Hospital of Sun Yat-Sen University. The register number is [2021]079. All participants should sign and informed the consents before ultrasound examination, and the Helsinki declaration was considered [22]. To maintain the quality of the report, this study was conducted according to the STROBE checklist [23] and also referred to the CONSORT checklist to some extent [24].

### 2.2. Participants

We recruited participants from April 2021 to January 2022 via advertisement posted at the rehabilitation department of the First Affiliated Hospital of Sun Yat-Sen University. The NCLBP group was enrolled based on the following criteria: the participants should meet the medical diagnostic standards for NCLBP [4], aged 18∼59 years with low back pain between the twelfth rib and the gluteus sulcus, pain intensity between 2∼5/10 according to the numerical rating scale (NRS) [13, 15, 25], and symptoms lasting for at least 3 months. For the healthy control (HC) group, participants were matched with the NCLBP group for demographic data such as sex, educational status, height, weight, age, and so on and with no symptom of low back pain.

The exclusion criteria were considered as follows. First, participants with smoking and chronic respiratory diseases (bronchial asthma, tuberculosis, chronic obstructive pulmonary disease, etc.) were excluded. Next, we excluded participants who had a history of spine or thoracoabdominal surgery and experienced kinesiotherapy frequently in the past 3 months [13]. In addition, participants with pregnancy and body mass index (BMI) > 31 kg/m^2^ were eliminated [26]. Finally, we excluded participants who had conditions that made them unable to cooperate with the examination (cognitive disorder, psychosis, current self-harm or suicidal ideation, major depression or anxiety, etc.) [25].

### 2.3. Instruments and Measures

The diaphragm thickness and excursion were examined by an experienced ultrasound doctor through a high-property ultrasound equipment (KONICA MINOLTA, SONIMAGE HS1, Tokyo, Japan). The examination methods and skills were carried out according to the consensus and expert recommendations of the European Society of Intensive Care Medicine [27].

**Figure 1:**
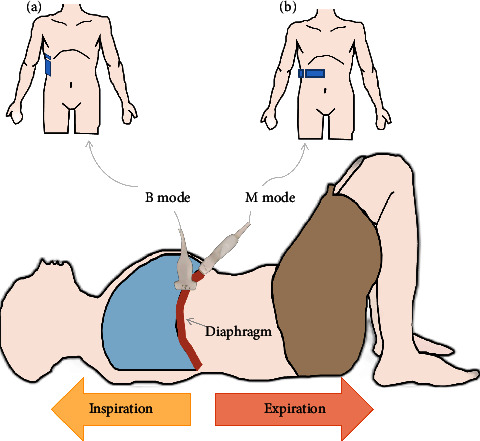
Sketch map of diaphragm measurement (a) Diaphragm thickness measurement (b) Diaphragm excursion measurement.

**Figure 2:**
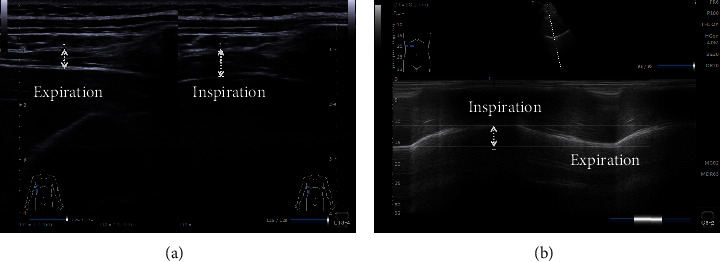
Ultrasound measurements of diaphragm. (a) Diaphragm thickness in B-mode. (b) Diaphragm excursion in M-mode.

We chose a linear array high-frequency probe (LINER PROBE, L18-4, 18 MHz) to assess the diaphragm thickness. The subjects laid supine on the examination bed and the ultrasound probe was located in the anterior axillary line between the subject's 8∼9 ribs (Figure 1(a)). The three parallel tissues were clearly visible in 2-dimensional B-mode ultrasound consisting of two hyper-echoic pleural and peritoneal layers and an intermediate muscle layer. The distance between the pleural and peritoneal layers was the thickness of the diaphragm which was measured at the end of deep inspiration (*T*^ins^)/expiration (*T*^exp^) in our research, respectively [16] (Figure 2(a)). The diaphragm thickness change rate (*T*^rate^) was computed by the formula: *T*^rate^ = (*T*^ins^ − *T*^exp^)/*T*^exp^*∗*100% [20, 28].

We selected a curved array of low-frequency probe (CONVEX PROBE, C5-2, 4 MHz) to evaluate the diaphragm excursion during deep breathing. We placed the probe at the bottom edge of the low rib cage between the anterior axillary line and the midclavicular line (the probe could also be slid from the navel to the lower right edge of the low ribs, with a higher rate of gain across the liver incision diaphragm) (Figure 1(b)). The highlighted diaphragm was legibly visible in B-mode window, and the movement of diaphragm could be easily obtained during the breathing cycle. Subsequently, we chose a measurement line to make the ultrasound beam perpendicular to the diaphragm under M-mode ultrasound which could evidently reveal the diaphragm excursion, moving the probe downward when inhalation, and the opposite when exhalation. The vertical distance between the highest plane and the lowest plane of the curve was the degree of diaphragm excursion [29, 30] (Figure 2(b)).

During the ultrasound examination, the participants were instructed to control their breathing. They need to breathe as deeply as possible and then exhale as slowly and completely as needed during the deep breathing. Also, subjects were verbally encouraged at each measurement.

### 2.4. Bias

We introduced the details of the examination to participants for better cooperation and to minimize the measurement bias. The ultrasound examiner was blinded to group allocation to reduce potential bias. Moreover, in order to reduce the error, we used the same measurement method, evaluated the diaphragm thickness and excursion 3 times, and took the average value as the final statistical measurement results.

### 2.5. Sample Size Calculation

The sample size calculation was accomplished by *G∗*Power (version 3.1.9.4). Six individuals with NCLBP (2.43 ± 0.56 mm) and six healthy subjects (2.85 ± 0.50 mm), considering the mean difference variables and standard deviation (MD ± SD) of* T*^exp^ was obtained between two groups in the pilot study. Then, two-sided alternative hypothesis, effect size of 0.79, an alpha of 0.05, a power of 0.80, and the allocation ratio (N2/N1) of 1 were inputted to compute the sample size. In this regard, 27 subjects in each group were needed for this study. Considering a 20% dropout rate, the total sample size was approximately 64 and 32 subjects per group.

### 2.6. Statistical Analysis

All data were analyzed by SPSS 20.0 (SPSS Inc., Chicago, IL, USA), and the results were considered statistically significant when the *P* value was lower than 0.05.

We first tested normality of the continuous variables data. Subsequently, the results were expressed as mean ± standard deviation (MD ± SD) and tested by independent-sample *t*-test when the data distributions fit normal curve (*P* ≥ 0.05) such as the indexes of age, weight, BMI, *T*^inx^, *T*^rate^, and *T*^exc^. When the data did not fit a normal curve (*P* < 0.05), results were expressed as median (interquartile range: 25%–75%) and tested by rank sum test (Mann–Whitney *U* test) such as the indexes of height and *T*^exp^.

For categorical data, the indexes of sex/pain duration time were tested by the chi-square test (*χ*^2^) and education level was tested by Fisher's exact test to calculate the statistics of the two groups.

Binary logistic regression analysis was used to analyze the possible factors for the occurrence of NCLBP after considering the multiple collinearity problem. The method of electing variables in the equation was based on toward to (toward: LR) maximum likelihood estimation. Age, sex, BMI, height, weight, *T*^ins^, *T*^exp^, *T*^rate^, and *T*^exc^ were included as independent variables, and NCLBP (yes/no) was included as a dependent variable. The significance of associations was evaluated at *P* < 0.05 with 95% confidence interval (CI) and odds ratio (OR).

Besides, the intraclass correlation coefficient (ICC) was obtained through reliability analysis (two-way mixed, absolute agreement, 95% confidence interval), the standard error measurement (SEM) was calculated as SEM = SD × $\sqrt{\left(1-ICC\right)}$, and the minimum detectable change (MDC) was calculated as MDC = 1.96 × $\sqrt{2}$ × SEM according to previous study [31, 32].

## 3. Results

### 3.1. Demographic Characteristics

Sixty-three participants (31 subjects in the NCLBP group and 32 subjects in the HC group) met the criteria and were finally enrolled. The baseline characteristics such as sex, age, weight, height, BMI, and education level had no significant differences between the two groups. The details are illustrated in Table 1.

### 3.2. Outcome of the Ultrasound Measured Parameters

**Figure 3:**
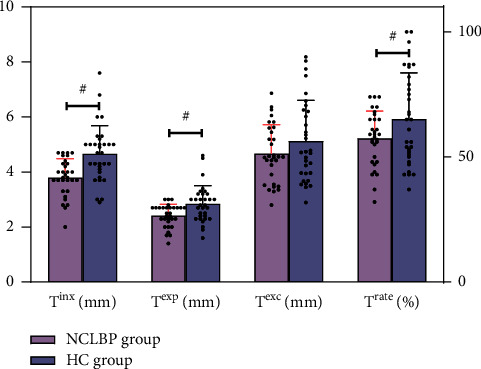
The dispersion graph of coordinate distribution for each indicator between two groups. ^#^*P* value of less than 0.05.

Compared with the HC group, the indexes of *T*^ins^, *T*^exp^, and *T*^rate^ in the NCLBP group decreased in varying degrees, and the differences were statistically significant (*T*^ins^ (*t* = −3.90, 95% CI = −1.30∼−0.42, *P* < 0.001), *T*^exp^ (*Z* = −2.79, *P*=0.005), *T*^rate^ (*t* = −2.03, 95% CI = −0.15∼0, *P*=0.047)). However, the index of *T*^exc^ did not show statistically significant difference (*t* = −1.42, 95% CI = −12.15∼2.09, *P*=0.161). The details are illustrated in Table 2. In addition, the dispersion graphs of the coordinate distribution for each indicator are shown in Figure 3.

### 3.3. Binary Logistic Regression Analysis of the Factors for the Occurrence of NCLBP

The binary logistic regression analysis results showed a statistically significant effect of *T*^rate^ (OR = 16.038, *P*=0.014, 95% CI 2.815∼9138.639) as well as *T*^exp^ (OR = 7.71, *P*=0.004, 95% CI 1.95∼30.49) on the occurrence of NCLBP. The rest of the independent variables (BMI, age, height, etc.) did not show a statistically significant effect. The details are illustrated in Table 3.

### 3.4. Intrarater Reliability

In view of three repeated diaphragm assessment of the subjects by the same ultrasound doctor, we calculated the intrarater reliability, and the results showed the following: *T*^ins^ (ICC = 0.940; 95% CI = 0.911∼0.961; Cronbach's a = 0.979; SEM = 0.011; MDC = 0.031), *T*^exp^ (ICC = 0.906; 95% CI = 0.862∼0.930; Cronbach's a = 0.967; SEM = 0.036; MDC = 0.101), and *T*^exc^ (ICC = 0.964; 95% CI = 0.946∼0.977; Cronbach's a = 0.988; SEM = 0.222; MDC = 0.615), respectively.

## 4. Discussion

In this study, the diaphragm thickness and excursion were examined by ultrasound in young and middle-aged subjects with and without NCLBP. We found that (1) the level of *T*^ins^, *T*^exp^, and *T*^rate^ decreased significantly in the NCLBP than HC group (*P* < 0.05); (2) in terms of *T*^exc^, although no statistical difference was seen between the two groups (*P* > 0.05), we still found that the *T*^exc^ index in the NCLBP group decreased to a certain extent, compared to the HC group; (3) the binary logistic regression analysis showed that the indexes of *T*^rate^ and *T*^exp^ were risk factors of NCLBP, implying that abnormal *T*^rate^ and *T*^exp^ might be conductive to the occurrence of NCLBP.

The diaphragm morphological structure and function are often reflected by measuring diaphragm thickness, its change rate, and excursion during inhalation and exhalation. Although the diaphragm changes rhythmically with breathing, the ultrasound evaluation of the diaphragm still has high accuracy and consistency [16, 33, 34]. This study also found high intrarater reliability (ICC varied from 0.906 to 0.964) which was in line with the other studies [35, 36]. In addition, we found that the total standard deviation of *T*^ins^, *T*^exp^, and *T*^exc^ values (0.967, 0.590, and 14.241, respectively) of the two groups was greater than SEM (0.011, 0.036, and 0.222, respectively) and MDC (0.031, 0.101, and 0.615, respectively). In this regard, the present study presents a good reliability according to the standpoint of Bland and Altman [37].

The diaphragm thickness could be obtained through B and M mode ultrasound, and both of the two modes have high accuracy and reproducibility [27, 38, 39]. Taking into account the fluid interfering effect under M-mode [38], the present study used B-mode ultrasound to measure the diaphragm thickness, which was in line with the studies by Ziaeifar et al. [16], Sarwal et al. [20], and Calvo-Lobo et al. [21]. The diaphragm thickness change rate (*T*^rate^) could reflect the true diaphragm function and work efficiency [40, 41]. No change or insufficient change of the diaphragm during breathing is an important manifestation of diaphragm paralysis or functional imbalance.

A recent study indicated that participants with NCLBP have a significant decrease of right diaphragm thickness change and thickness at expiration [16]. Another study manifested that athletes with lumbopelvic pain have less bilateral diaphragm thickness and lower right diaphragm change rate during inspiration than healthy paired athletes [21]. Consistent with the results of the above research, we also detected that the diaphragm thickness and diaphragm thickness change rate were reduced significantly in the NCLBP group, compared with HC group.

Diaphragm excursion is another important component of diaphragm function detection. It is an indicator to assess whether the body could inhale enough gas to meet the needs during each inspiration, which could reflect the lung ventilation capacity. The intrarater reliability of the diaphragm excursion measurement by M-mode ultrasound in this study was consistent with Mohan et al.'s study [42], which reported excellent ICC values (0.964 and 0.92, respectively), but inconsistent with Gram et al.'s study (ICC values range from 0.65 to 0.69) [43]. The reason for this distinction between our study and Gram et al.'s study might be due to different interval measurement periods.

Our study found that the diaphragm excursion was somewhat reduced but had no statistical difference in the NCLBP group compared to the HC group, which was consistent with the findings of Ziaeifar et al. [16] and Calvo-Lobo et al. [21]. Inconsistent with the present study, a cross-sectional study regarded that the diaphragm excursion and respiratory muscle endurance were lower in the NCLBP group [15]. Considering that the diaphragm excursion is almost greater in males than in females [30, 44], the reason for this discrepant results between our study and previous study might be due to gender differences (2 males and 66 females in Mohan et al.'s study [15] versus 30 males and 33 females in the present study). In addition, studies examining the diaphragm by MRI had found that the range of diaphragm excursion in the NCLBP group was less than that in the HC group [11, 45], which was also different from our study. There are several reasons for this discrepancy. On the one hand, the movement changes in each part of the diaphragm could be dynamically detect by MRI. However, in the present study, ultrasound only examines the posterior diaphragm activity [16, 46, 47]. On the other hand, the M-mode ultrasound measures the intersection movement of the sampling line between the diaphragm and evaluates the diaphragm movement contraction at a certain point, rather than the overall assessment, which is probably not as sensitive as MRI. Consequently, we considered that the result bias might be caused by different measurement methods (MRI versus ultrasound), as also described by Ziaeifar et al. [16].

Based on our study, the binary logistic regression analysis elucidated that the reduction of *T*^rate^ increases the risk of NCLBP occurrence, which was supported by another study showing that the right diaphragm thickness change might be conductive to the occurrence of NCLBP [16]. Moreover, the present study also found that *T*^exp^ might be a risk factor for the occurrence of NCLBP, which has not been reported previously [15, 16]. Former study identified that diaphragm change rate, as an indicator of respiratory function, could reflect the strength of the diaphragm contraction [14]. In addition, patients with chronic low back pain have weakened abdominal muscles [48, 49], reduced trunk stability and intra-abdominal pressure [50], and uncoordinated movement [51, 52]. The abnormal regulation of intra-abdominal pressure may play a pivotal role in process of NCLBP occurrence. Moreover, patients with chronic low back pain might be associated with respiratory pattern disorder and diaphragm dysfunction [53, 54]. Considering the above views, it is reasonable to believe that diaphragm dysfunction might be associated with the occurrence of low back pain, and the role of the diaphragm could be strengthened during the treatment and reeducation of it, with particular attention to expiratory function.

The present study still has several limitations. Firstly, since only young and middle-aged groups were included in this study, other age grades of NCLBP shall be explored in the future work. Secondly, considering the right diaphragm was more accessible than the left, we only examined the right diaphragm. Bilateral detection of diaphragm may be conductive to comprehensive understanding of the role of diaphragm morphological structure and function changes in subjects with NCLBP. Thirdly, because the affected side (unilateral or bilateral) of NCLBP was not distinguished, the compensation of the healthy side to the pain side might bias the results [55, 56]. Lastly, due to the lack of functional assessment related to postural control, the potential relationship between the diaphragm and postural control is part of our further investigation.

## 5. Conclusion

This study assessed the diaphragm by using ultrasound and found that the young and middle-aged subjects in NCLBP group had a smaller diaphragm thickness at the end of inspiration/expiration and diaphragm thickness change rate during deep breathing, compared with the healthy control group. In addition, the abnormal diaphragm thickness change rate and diaphragm thickness at the end of expiration were potential risk factors of NCLBP occurrence. Although there are limitations in this study, it still has certain clinical significance. This study could provide new insight for the pathogenesis of NCLBP and suggests diaphragm morphological structure and function as a potential approach for assessment and rehabilitation treatment for NCLBP.

## Figures and Tables

**Table 1:** Baseline characteristics of subject's data.

	NCLBP group (*N* = 31)	HC group (*N* = 32)	*t*/*χ*^2^/*Z*	*P* value	95% CI
Age (years)	30.51 ± 6.05	29.13 ± 5.68	−0.94	0.350^a^	(−1.56∼4.35)
BMI (kg/m^2^)	21.57 ± 2.55	22.27 ± 3.20	−0.96	0.343^a^	(−2.16∼0.76)
Height (cm)	167.00 (162.00–173.00)	166.5 (160.00–176.00)	−0.220	0.826^b^	NA
Weight (kg)	60.13 ± 9.40	63.18 ± 12.49	−1.10	0.278^a^	(−8.64∼2.52)
Sex (*n*, %)					
Male	14 (45)	16 (50)	0.15	0.701^c^	NA
Female	17 (55)	16 (50)			NA
Education level, *n* (%)					
Junior	0	0			
Senior	2 (6.5)	0 (0)		0.238^d^	NA
College or higher	29 (93.5)	32 (100)			
Pain duration, *n* (%)					
3 months to 1 year	8 (25.8)	NA			
1 year to 5 years	21 (67.7)	NA			NA
5 years to 10 years	1 (3.2)	NA			
Over 10 years	1 (3.2)	NA			

^a^ *t*-value;^b^*Z*-value;^c^*χ*^2^-value; ^d^Fisher's exact test.

**Table 2:** Comparison of the index of *T*^ins^, *T*^exp^, *T*^rate^, and *T*^exc^ between the two groups.

	NCLBP group (*N* = 31)	HC group (*N* = 32)	*t*/*Z*	*P* value	95% CI
*T* ^ins^	3.80 ± 0.68	4.66 ± 1.02	−3.90	<0.001^a#^	(−1.30∼−0.42)
*T* ^exp^	2.50 (2.20–2.70)	2.80 (2.32–3.20)	−2.79	0.005^b#^	NA
*T* ^rate^	0.57 ± 0.11	0.65 ± 0.18	−2.03	0.047^a#^	(−0.15∼0)
*T* ^exc^	51.37 ± 11.57	56.40 ± 16.23	−1.42	0.161^a^	(−12.15∼2.09)

^a^ *t*-value; ^b^*Z*-value; ^#^*P* value of less than 0.05.

**Table 3:** Binary logistic regression analysis of NCLBP factors.

Dependent variable	Independent variable	*B*	S.E.	Wald	*P*	OR	95% CI
NCLBP (yes/no)	BMI	−0.244	1.023	0.057	0.811	0.783	0.105∼5.823
	Height	−0.016	0.074	0.044	0.834	0.985	0.851∼1.139
	Weight	0.012	0.031	0.151	0.698	1.012	0.952∼1.077
	Age	−0.023	0.053	0.185	0.667	0.977	0.880∼1.085
	Sex	−0.674	0.679	0.984	0.321	0.510	0.135∼1.929
	*T* ^exc^	−8.853	5.739	2.379	0.123	0.000	0.000∼10.983
	*T* ^ins^	−8.820	5.631	2.453	0.117	0.000	0.000∼9.177
	*T* ^rate^	5.078	2.063	6.060	0.014^#^	16.038	2.815∼9138.639
	*T* ^exp^	2.043	0.701	8.490	0.004^#^	7.714	1.952∼30.485

^#^ *P* value of less than 0.05.

## Data Availability

The data used to support the findings of this study are available from the corresponding author upon reasonable request. (Corresponding email; wangchuh@mail.sysu.edu.cn).

## Supplementary Materials

Supplementary Materials Supplementary 1: STROBE checklist; Supplementary 2: CONSORT checklist.
